# Quantitative Tools for Examining the Vocalizations of Juvenile Songbirds

**DOI:** 10.1155/2012/261010

**Published:** 2012-06-04

**Authors:** Cameron D. Wellock, George N. Reeke

**Affiliations:** Laboratory of Biological Modeling, The Rockefeller University, 1230 York Avenue, New York, NY 10065, USA

## Abstract

The singing of juvenile songbirds is highly variable and not well stereotyped, a feature that makes it difficult to analyze with existing computational techniques. We present here a method suitable for analyzing such vocalizations, windowed spectral pattern recognition (WSPR). Rather than performing pairwise sample comparisons, WSPR measures the typicality of a sample against a large sample set. We also illustrate how WSPR can be used to perform a variety of tasks, such as sample classification, song ontogeny measurement, and song variability measurement. Finally, we present a novel measure, based on WSPR, for quantifying the apparent complexity of a bird's singing.

## 1. Introduction

A bird's song can be a powerful marker of identity, used by other birds—and humans—to identify the singer's species or even to identify a single individual. In many species this song is innate, but for the Oscine songbirds, every bird must acquire its own song [1, 2]. With one such bird, the zebra finch (*Taeniopygia guttata*), it is the males that sing, and juvenile males learn their song from nearby adults such as their father [3]. The learning process has two overlapping but distinct parts: in the first, the animal hears the songs of other birds and somehow commits to memory a model of the song it will sing; in the second, the animal learns how to produce a version of this memorised song through practice [1].

**Figure 1:**
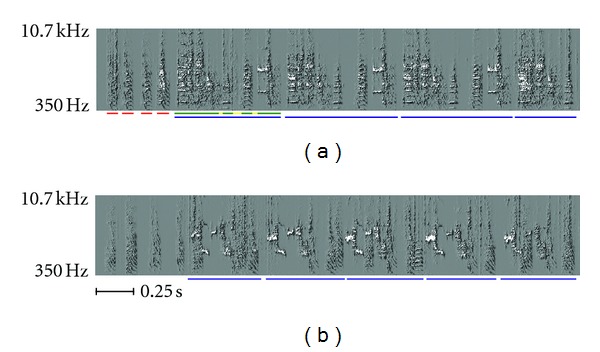
(a) Spectrogram of a bout of singing from an adult zebra finch. Noted in the figure are the following song parts: introductory notes, underlined in red; syllables, underlined in green; the silent interval between syllables, underlined in yellow. The blue lines mark the repetitions of the bird's motif. Note that each performance of the motif appears much like the others, except for the truncated final motif. (b) Spectrogram of a bout of singing from a different zebra finch. Although its song is also highly stereotyped, it is visibly different from the song of the bird featured in (a). For convenience, blue lines once again mark repetitions of the bird's motif.

As adults, zebra finches sing in bouts during which they perform their single song motif a variable number of times. The song motif of a zebra finch is on the order of one second long and is composed of multiple syllables, elements separated by silence or a sharp drop in amplitude. Syllables can often be broken down further into notes, segments of distinct sound quality. These notes may demonstrate pronounced frequency modulation and complex harmonics. Adult zebra finches typically exhibit a very high degree of stereotypy in their song, with one performance of the song's motif being very similar to any other. Two typical examples are shown in Figure 1.

In the early stages of a juvenile's song production, vocalizations tend to sound very little like the song of an adult, instead sounding more like a kind of babbling [4]. This earliest stage is called “subsong” [1]. From this, the juvenile progresses to a style of vocalization, “plastic song” [1], which is low in stereotypy but in which the precursors of adult-like sounds can be identified. Eventually, at approximately 80 days posthatch [5], the juvenile learns to produce its song with a high degree of stereotypy and its song-learning process is complete. For the zebra finch, this song will remain largely unchanged for the rest of the animal's life.

**Figure 2:**
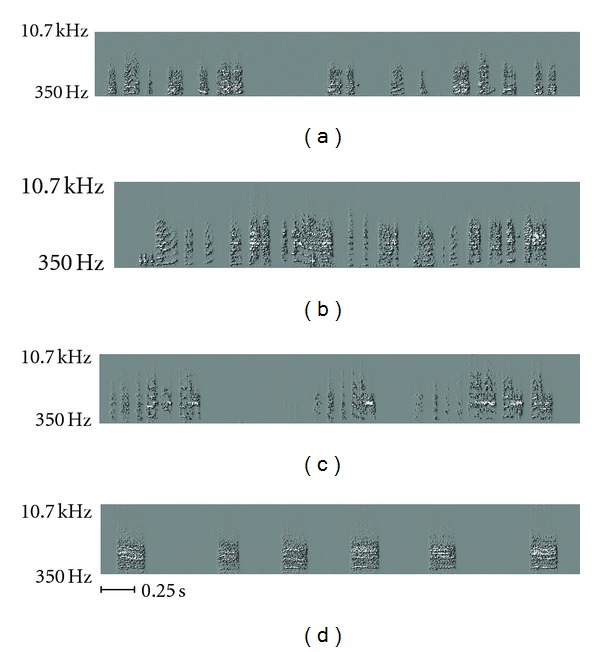
(a) Spectrogram of a juvenile's vocalizations produced during the babbling phase, at approximately 36 days posthatch. Note the general lack of stereotypy. (b) Spectrogram of a juvenile's vocalizations produced during the early plastic song phase, at 41 days posthatch. (c) Spectrogram of a juvenile's vocalizations produced during the plastic song phase, at 47 days posthatch. Although the sounds are more adult-like in terms of spectral profile, they still lack the stereotypy of adult birds. (d) Composite spectrogram of a series of calls from a juvenile zebra finch (40 days posthatch). By eye and by ear, these are easily differentiated from adult song.

Another class of vocalization is the “call,” which can serve multiple purposes [2]. Calls are typically short (200–500 ms) continuous sounds that might be described as “honk-like.” Zebra finches of both sexes, including juveniles, produce calls. Examples of juvenile song and calls are shown in Figure 2.

In the course of our research, we have at times wanted a tool to identify and compare juvenile vocalizations, primarily to assist in the sorting of large numbers of recorded samples. Although a number of tools exist to compare the songs of adult birds, we have found that, due to the low stereotypy of juvenile singing, these tools do not perform well on samples from juveniles.

The simplest method of comparing song samples is to calculate some measures of correlation between samples, either on their waveforms or spectrograms. This method is employed by several popular tools [6, 7] but works adequately only if the sounds being compared are very similar in timing, ordering, and tone. A related technique is dynamic time warping (DTW) [8], which can compensate for differences in timing but not ordering. DTW-based analyses can be performed on spectrograms or spectrogram-derived measures, such as cepstra [9]. Another strategy, used by at least one popular tool [10], might be described as heuristic feature analysis. A set of measures (e.g., peak frequency, frequency modulation, and spectral entropy) is used to characterise a sample, and these measures are used to compare two samples according to some set of criteria. Although these tools typically do not require the samples being compared to be highly similar, it has been our experience that, with juvenile vocalizations, these methods can produce similarity scores that vary greatly between pairs of samples that, to a human observer, appear more or less equally similar.

The key feature that all these existing methods have in common is that they are designed to compare one single sample against another single sample. For highly stereotyped adult birdsong, this approach makes perfect sense, but, for juveniles, it may not be appropriate: the high variability of juvenile song means that two samples from the same bird, taken seconds apart, may not be “similar” in any reasonable sense, and yet both are representative of that animal. With a large enough sample set, however, we should be able to identify all the characteristic sounds produced by a bird and be able to describe new samples in terms of how typical they are, even if the new sample does not seem particularly similar to any other sample.

Other methods for song analysis exist, such as the spectrotemporal modulation analysis used by Ranjard et al. [11], the rhythm analysis of Saar and Mitra [12], or the PCA-based feature analyses of Feher et al. [13]; however these methods as presented are unaware of syllable sequencing [11] or are very highly specialized [12] and are not suitable for general-purpose use.

In this paper, we present a new method for comparing a sample of juvenile birdsong against a model built from a set of training samples. We call this method windowed spectral pattern recognition (WSPR). This method provides a measure of typicality for comparing test samples to the training samples. We show that WSPR is effective as a classifier and may be better suited to this task than another popular tool. We also show that WSPR is relatively robust to changes in a key parameter. Lastly, we demonstrate that the models produced by WSPR can be used to provide measures of song ontogeny, stereotypy, and complexity

## 2. Methods

### 2.1. Housing and Care of Juvenile Zebra Finches

Audio recordings from three juvenile male zebra finches provided the data used in this paper. From hatching until 25 days posthatch, the juveniles were housed with their mothers, fathers, and clutch mates in a family setting. From 25 days to 35 days, the juveniles were housed in small cohorts of 2–4 individuals along with an adult tutor. From 35 days to between 50 and 60 days, the juveniles were housed singly in auditory isolation chambers. At all times the juveniles were given food and water *ad libitum*. The juveniles were cared for in accordance with the standards set by the American Association of Laboratory Animal Care and Rockefeller University's Animal Care and Use Committee.

### 2.2. Recording of Juvenile Birds and Manual Identification of Samples

Continuous recordings were made of three isolated juvenile male zebra finches from 35 days posthatch to 60 days posthatch with Behringer ECM-8000 measurement microphones (Behringer International GmbH, Willich, Germany) and Rolls MP13 preamplifiers (Rolls Corporation, Murray, UT). A MCC PCI-DAS6013 digital acquisition card (Measurement Computing Corporation, Norton, MA) was used to digitise the audio inputs. Recordings were made at 44.1 kHz, 16 bits/sample, and stored as lossless FLAC [14] files.

We examined recordings with Audacity sound editing software [15] and manually identified vocalization bouts as being calls, song, or neither. Vocalization bouts identified as calls or song were eliminated if they contained excessive levels of spurious noise—flapping of wings, footfalls on metal bars, and the like—or if they were less than one second long. 2026 samples were taken from the three birds. Each bird's samples were assigned to one of four different sample sets: song training, song testing, call training, and call testing.

### 2.3. Building a Model and Scoring Using WSPR

A test model was built using the WSPR command-line tool from a combination of both training sample sets, using the parameters given in Table 1. During the clustering phase of the WSPR algorithm, the set of spectra that was clustered as well as their cluster assignments were extracted and silhouette statistics [16] were computed using the “cluster” package [17] for the *R* statistical computing environment [18]. For comparison, a random dataset was also generated and clustered. A set of 7 500 vectors, each the same length as the WSPR spectra, was produced, with every value in each vector being a randomly generated number from a [0,1] uniform distribution. This random dataset was clustered using *R*'s “*k* means” function, and silhouette statistics were computed as for the clustered spectra.

### 2.4. Binary Classification of Juvenile Vocalization Samples

For each bird, a binary classifier was constructed using the WSPR algorithm for classifiers described in the appendix. The classifier contained one model for song, built from the song training samples, and one for calls, built from the call training samples. The parameters used in the construction of these models are found in Table 1.

All testing samples were presented to the classifier. Samples were assigned to a group by the classifier, and the Matthews correlation coefficient (MCC) [19] was used to assess the accuracy of the assignments. The number of samples used as training and testing data for each bird, as well as mean sample lengths, is given in Table 2.

For comparison, Sound Analysis Pro+ (SA+) [10] was also used to classify samples from the first bird. From the 669 original samples, two hundred were randomly chosen, with fifty from each of the four sample sets (song training, song testing, call training, and call testing). The samples were loaded into the SA+ software and run in a series of pairwise comparisons using SA+'s “batch similarity” tool, so that each test sample was compared against one training sample from the “call” set and one training sample from the “song” set. SA+'s volume threshold was reduced, but otherwise was run with all settings at their default values. The calculated similarity scores were then exported from SA+ for statistical analysis.

When used as a classifier, the same classification method described in the appendix (Classification Using Multiple Models) was used on the SA+ scores, with the exception that the SA+-generated scores were used in place of WSPR's raw scores.

### 2.5. Measuring Song Ontogeny and Stereotypy

Recordings from the juveniles examined previously were taken, and, for each bird, two models were made: an early model, consisting of the earliest 100 song samples; a late model, consisting of the latest 100 song samples. The remaining samples from each bird were grouped by day and scored against the models.

For one juvenile, all samples were grouped into blocks of five consecutive days each, and models were generated for each group, and the standard error of the nonstandardized scores samples used to build the model against the model was calculated.

### 2.6. Testing the Effects of Parameter Selection on Score Distributions

Fifteen models were built with varying numbers of prototypes: 10,20,…, 150. All models were built using the same set of song training data for the first bird as described previously. Except as noted in the results, the WSPR parameters are found in Table 1. Each sample from the first bird's song test data was scored against all 15 models. Means and standard deviations were calculated for the scores from each model.

### 2.7. Estimating the Stereotypy and Complexity of Sample Sets

Additional recordings were made of an adult zebra finch, over 100 days old, with equipment and conditions identical to those used for the juvenile recordings, except that the DAQ digitiser was bypassed and the computer's built-in audio input was used instead. One hundred samples of adult song were manually identified and extracted from the recordings. For each of the three juvenile birds, the WSPR algorithm was used to generate separate models for song and calls on all available samples, including both training and testing data from the earlier experiments. For the adult bird, a model was generated for its song on the 100 collected samples. For all models, all samples were concatenated and the combined samples were truncated to a length of exactly two million audio samplings (approximately 45 seconds); each model was built from its corresponding concatenated sample. All samples were scored against the models they were used to train, and the standard deviations of all scores against each model were calculated. The models were generated using the parameters found in Table 1, with the following exceptions: STFT window width, 4096 samples; STFT step size, 1024 samples; model prototypes, 50; model window width, 25. The WSPR complexity of each model was also calculated according to the algorithm found in the appendix (Calculating the Complexity of a Model).

## 3. Results

### 3.1. Building a Model Using the WSPR Algorithm

**Figure 3:**
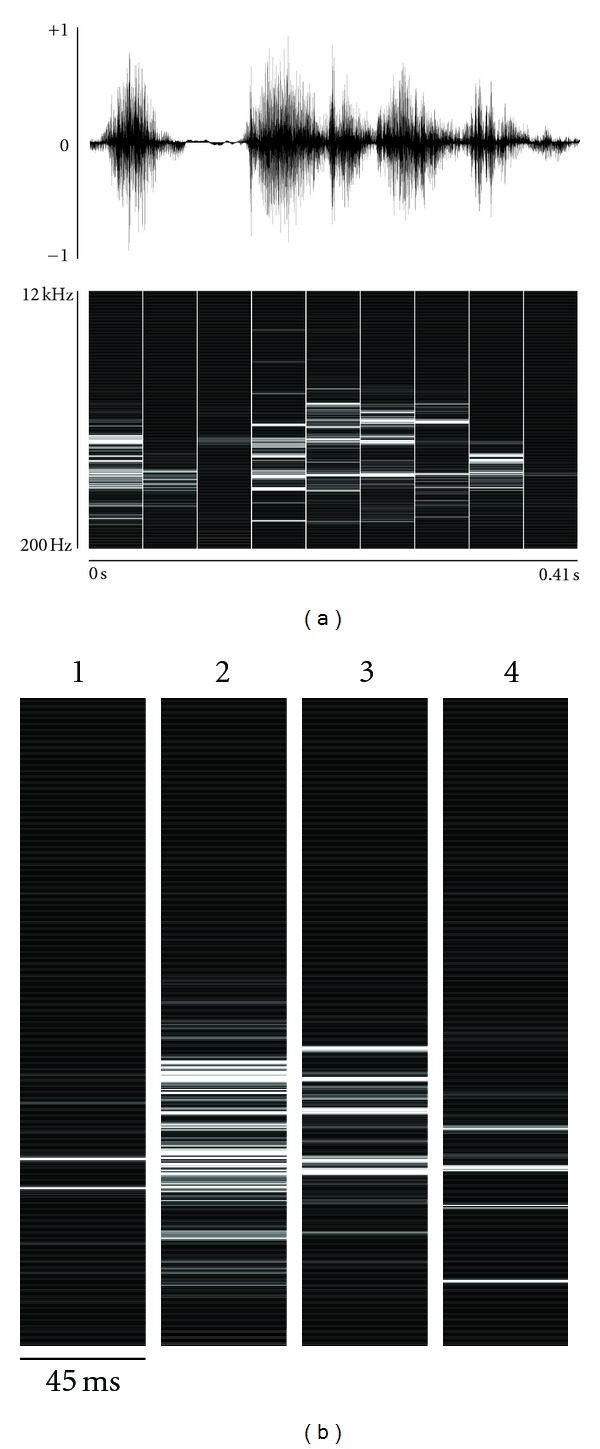
(a) Using a discrete Fourier transform, sounds are converted from waveform (top pane) to a sequence of frequency spectra (bottom pane)—in essence, a spectrogram. Note that each discrete frequency spectrum accounts for a period of time much larger than the sampling rate; the effect is exaggerated here to make this clear. (b) Examples of possible prototypes. In the WSPR algorithm, every segment of sound will be matched to a similar prototype and coded as the prototype's index number (1, 2, 3, 4, etc.)

Model building is composed of two discrete steps: creating an encoding and producing tables of observed frequencies of patterns. To create an encoding, a set of 100 samples of juvenile plastic song was taken from a single individual. Samples were converted from digitized waveforms into a frequency-versus-time representation (a spectrogram) using a discrete-time Fourier transform (DTFT), as illustrated in Figure 3(a).

From the set of all training samples' spectrograms, 7 500 spectra were chosen without replacement. These were clustered using a *k*-means clustering algorithm [20] into 120 clusters. The *k*-means clustering algorithm works to divide the 7 500 spectra into *k* clusters, with all the items in each cluster more similar to each other than to the members of any other cluster. Each cluster represents a single kind of “sound” that the bird makes: clusters may represent single notes, harmonic stacks, staccato bursts, or other types of sound. The members of each cluster were averaged to produce a set of prototypical sounds, one prototype per cluster, and each prototype was assigned a unique index number (its “symbol”); these prototypes formed the basis for the encoding. Sample prototypes can be found in Figure 3(b).

The silhouette statistic [16] was used to characterize how well the clusters divided the underlying spectra. The silhouette statistic is a unitless value between −1 and +1; a silhouette value of 1 implies that an item is ideally clustered, a value of −1 implies that an item should be assigned to another cluster, and a value of 0 implies that an item could just as easily be assigned to another cluster as to its current cluster. For the clusters used to produce the prototypes, the mean silhouette value was 0.264. In contrast, the mean silhouette value for a randomized dataset was 0.0252. This suggests that many clusters are only moderately separated from their neighbors, which is reasonable given the large number of clusters and the high variability of the underlying bird vocalizations.

Sounds were encoded by first converting from waveform to frequency-versus-time representation, as before. Each discrete frequency spectrum was compared to the full set of prototypes, and the spectrum was encoded as the index number of the prototype it was most similar to (determined by root mean square deviation). Each sample was thus converted from a waveform, to sequence of frequency spectra, to a sequence of symbols. With this, the encoding step of building a model was completed. The average sample was 2.42 seconds long; once encoded, the average sample was 1063 symbols long.

The second part of the model-building process is the one in which patterns in the bird's song are identified. A window width (*w*) of 11 was set, and an anchor position (*a*) of 6 was calculated. An array of dimension 120 × 11 × 120 was created; all values in the array were set to zero. A count was tallied of the number of times symbol *y* was seen at position *z*, given that symbol *x* was seen at position *a*, for all *x*, *y*, and *z*, by scanning each sample and tallying the observed symbols.

### 3.2. Scoring a Sample

A single test sample was first encoded using the same encoding method described for model building. After encoding, the sample was scanned over in a manner very similar to how the frequency array was built; however, instead of modifying the array, the values in the array were incorporated into a score, so that more common sequences of symbols will score higher than less common ones. The exact formula used is described in the appendix. Once the nonnormalized (raw) score was generated, it was standardized (as a *z*-score) in order to make the scores easier to interpret. The standardization procedure is also described in the appendix. A single arbitrary sample produced a raw score of 0.34, a *z*-score of 0.45, and a *P* value of 0.32, implying that the sample was fairly typical of the model's training data, which in this case was to be expected, as the test sample was identified by the authors as being qualitatively “of a kind” with the training data.

### 3.3. Binary Classification

The motivation for developing this method was to quickly classify very large sets of recorded samples, so it seemed fitting to examine its fitness for this purpose.

In the authors' recording setup, juveniles were recorded continuously, twenty-four hours a day. It was not possible to listen to all of this audio—indeed, for months, recordings were being accumulated much faster than a single person could listen to, even if that person listened to them every minute of every day.

A simple amplitude threshold check was able to eliminate most of the recordings; however, this still left tens of thousands of audio events—samples—that needed to be examined. One of the primary goals in developing the WSPR tool was to create a reasonably robust tool that could sort through such large sample sets in minutes or hours, rather than days, and further reduce the amount of work that would need to be done manually.

A timing test was able to show that the WSPR algorithm was indeed suitable for use with such large datasets. A model was built from 200 samples; on a reasonably fast machine (Intel Core i7, 2.67 GHz clock speed), the model-building process took 12.1 seconds. Scoring 200 samples against that model took only 1.6 seconds. Assuming those 200 samples are representative of a larger set, it would take about 15 minutes to score 100 000 samples. By contrast, 200 pairwise comparisons were done using the SA+ program with the same sample set. These 200 comparisons took 593 minutes to complete on the same machine. Scaling up, comparing 100 000 sample pairs would take about 200 days to complete.

**Figure 4:**
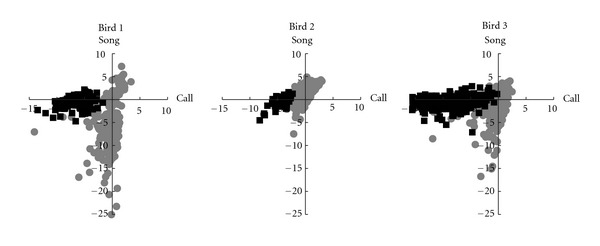
Performance on a classification task using standardized *z*-scores. Each test sample was scored against both “song” and “call” models. Gray points were manually assigned to the “call” class, while black points were manually assigned to the “song” class. For all birds, the two classes of sounds are well separated.

While speed is important, it is of little use if the results are inaccurate. To test WSPR's accuracy, a WSPR classifier was built comprised of two models, one of “call” samples, and one of “song” samples.  Figure 4 shows the raw scores against both “call” and “song” models for the test data in scatter plots. The MCCs for the classifications of each bird's samples were 0.93, 0.75, and 0.65, with a cumulative MCC of 0.78.

**Figure 5:**
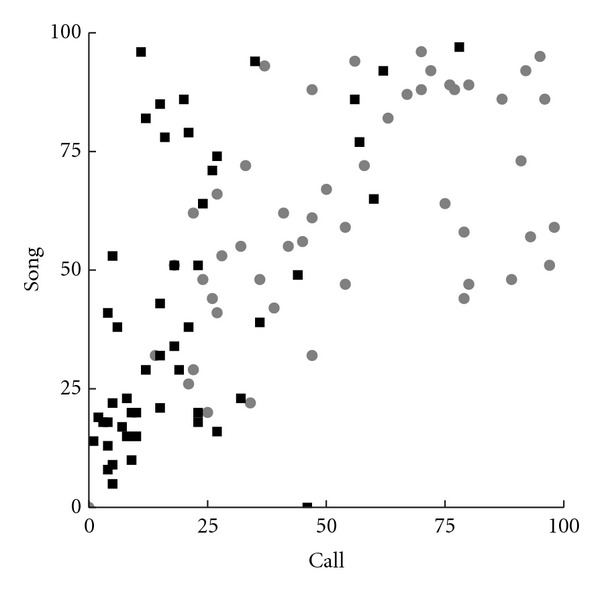
SA+ scores, performance on a classification task. Black points were manually assigned to the “song” class, and gray points were manually assigned to the “call” class.

It is also worth comparing the accuracy of WSPR classifications to SA+ scores.  Figure 5 shows the raw scores produced by the SA+ program. The MCC for the SA+-based classifier was 0.57, somewhat less than that for the WSPR classifier. On this task, WSPR made about 1/3 as many classification errors as SA+, although both produced fairly good results.

### 3.4. Song Ontogeny

In addition to its use as a classifier, the WSPR tool may also be useful for more analytical tasks. To that end, a test was devised in which WSPR was used to track the ontological development of three juvenile zebra finches.

To do this, once again two models were created for each finch, one from a set of early samples, near day 35, and one from a set of later samples, near day 50. Sets of intermediate samples were then taken, organized by day, and scored against each model. The difference between these two scores, specifically the late-model score minus the early-model score, indicates the extent to which the test sample was more typical of the late model than the early model.

**Figure 6:**
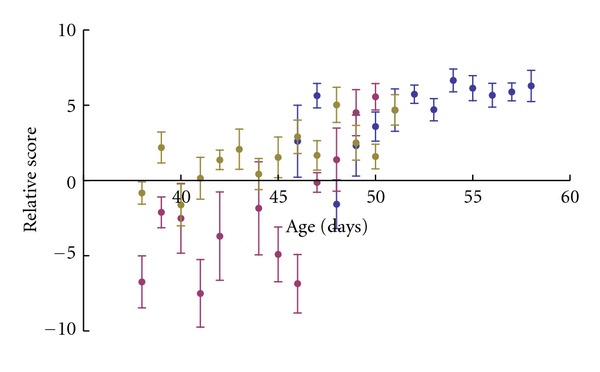
Measuring progress in song development. Each colour represents a different bird. For each bird, an early model and late model were built of the first and last 100 samples available; all other samples were compared against both models and their difference calculated, so that negative scores suggest a sample was more typical of the early model, and positive scores suggest a sample was more typical of the late model. Each point is the mean of all samples for that day, and error bars indicate standard error. All birds progress from being essentially early-like to being late-like, but unevenly and at different rates.

Figure 6 shows that the bird's songs do progress over time towards similarity with each bird's late model. According to the scores, the birds' songs develop unevenly at times and at different rates, an observation in accord with the authors' personal experiences.

WSPR might also be used to measure stereotypy, a task it seems well suited for given its emphasis on large sample sets. One simple and intuitive measure of stereotypy using WSPR would be the standard deviation or standard error of a sample set against a model; this is the measure used here. This measure is essentially one of variability: the lower the standard error, the greater the stereotypy.

**Figure 7:**
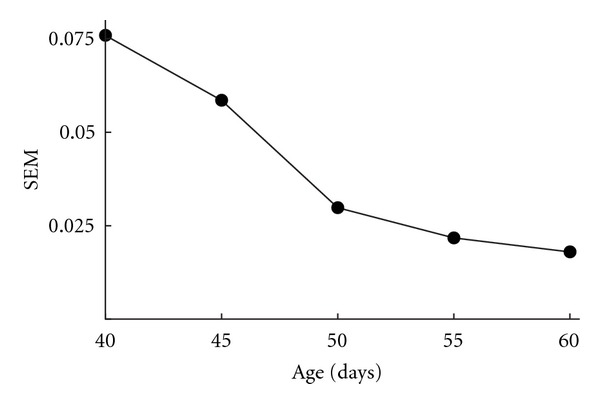
Nonstandardized score standard error as a bird develops its song. As the bird matures, the variability in its singing decreases.

The samples from one bird were grouped into five-day periods, and a model was built for each period. The samples were then scored against their models, and the standard error of the scores was used as a measure of apparent stereotypy.  Figure 7 shows the change in standard error for scores as a bird's song develops. As one would expect, variability decreases and stereotypy increases as the bird ages. There is a noticeable decrease in the rate at which variability declines around day 50.

### 3.5. Effect of Parameter Selection on Scores

It is important to know how sensitive the WSPR algorithm is to changes in parameters. There is a possibility that small changes in parameters might lead to large changes in scoring accuracy, a situation that would pose a practical problem for the use of the algorithm. There are three key parameters in the model that can be manipulated: the width of the STFT window, the number of prototypes, and the width of the model window.

Two of these parameters, the width of the STFT window and the width of the model window, are determined by the data being analyzed: for the width of the STFT window, the expected maximum length of time over which a sound would be approximately constant; for the width of the model window, the expected length over which patterns would be identifiable. As such, one would expect the scores to vary considerably as these parameters are changed; furthermore, the structure of the data should suggest ranges for these parameters.

There is therefore only one major parameter remaining that must be set in an ad hoc fashion: the number of prototypes. There are potential problems with having either too few or too many prototypes. If there are too few prototypes, sounds with qualitatively different spectral profiles will be assigned to the same prototype, the specificity of the encodings will fall, and the model may produce additional false positives. If there are too many prototypes, sounds that are qualitatively similar will be assigned to different prototypes and the number of false negatives produced will rise.

**Figure 8:**
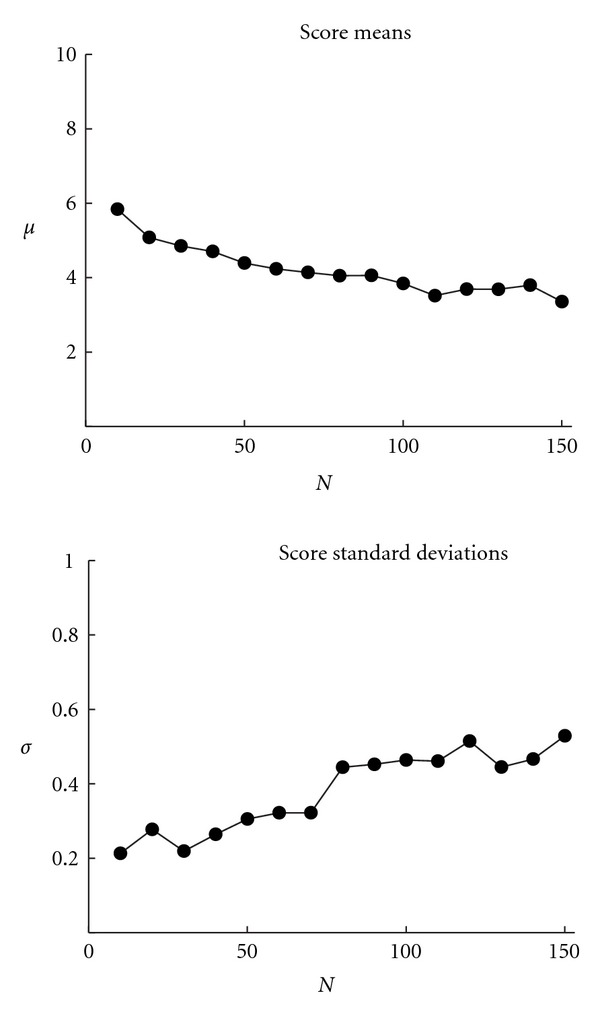
Scores and standard deviations as a function of the number of prototypes used (*N*). Scores were raw (nonstandardized). Neither scores nor standard deviations change abruptly in the face of small changes to the number of prototypes.

Figure 8 shows how the mean score and standard deviation of scores change in relation to the number of prototypes used to build the model. It can be seen that both the score means, and to a lesser extent, the standard deviations, level off when more than roughly 100 prototypes are used. This suggests that the method is insensitive to this parameter as long as sufficiently large set of prototypes is used.

### 3.6. Stereotypy and Complexity

Finally, there is the possibility of using the WSPR algorithm as a basis for measuring the complexity of a bird's song. Exactly what is meant by “complexity” in the context of birdsong is open to debate, but most researchers would probably agree it involves the number of distinct sounds an animal makes, as well as the patterns of those sounds. For many years, people have used informal measures of song complexity, such as the number of distinct notes or syllables in a song [21]. In addition to a high degree of subjectivity, these measures can be difficult to apply to birds with variable songs, such as juveniles or species that improvise when singing. As an alternative, WSPR can be used to generate a measure of complexity based on ideas from statistical complexity theory and information theory.

 A WSPR model contains a considerable amount of information about the sounds in a bird's repertoire and the likely sequences of sound it will produce—exactly the kind of information needed to measure complexity. Our method mines a WSPR model to produce a measure of the model's complexity, which is in turn a reflection of the complexity of the sample set used to build the model (see the appendix). This method was tested against vocalizations from a single bird as it learned to produce its song.

**Figure 9:**
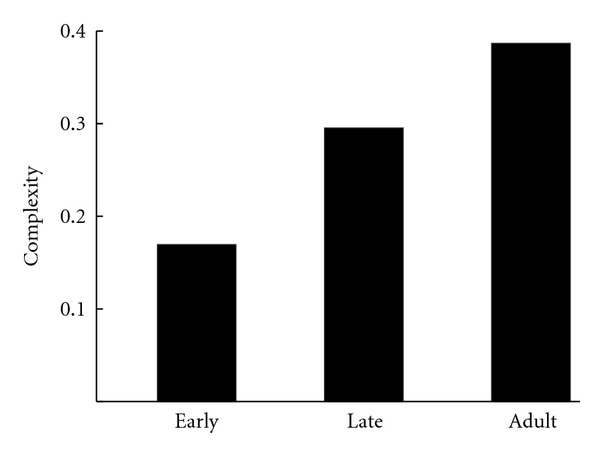
Complexity measured for early juvenile, late juvenile, and adult. As birds age, the apparent complexity of their song increases.

Figure 9 shows complexity scores for models built on early juvenile, late juvenile, and adult models. There is a marked rise in complexity as the bird's song develops, which coincides with intuitive expectations.

## 4. Discussion

### 4.1. About the Method

The WSPR algorithm attempts to identify recurring patterns in the vocalization samples submitted as training data. It does not need a high degree of top-level similarity, nor does it look for any particular features. At its heart, the algorithm is built upon a simple expression of conditional probability: given that at this moment the sample sounds like *x*, what are the probabilities of the sounds heard in the preceding and following moments?

WSPR breaks the sample into short segments and, using spectral analysis techniques, identifies the significant frequency components of each segment. It then estimates how probable such a frequency profile might be and how probable its neighbouring profiles are. The probability estimates are based on the distributions observed in the training data.

### 4.2. On the Use of *k*-Means Clustering to Divide Data

How well does *k*-means clustering divide the data? The mean silhouette statistic calculated for our initial model was 0.264, suggesting that the underlying data clusters only moderately well. The motivation however for using *k-*means clustering here was not to identify clusters but to discretize the data in a reasonably natural way, to the extent that natural partitions may exist within the data. If natural partitions do not exist, the data will simply be divided into neighboring discrete regions. Other partitioning strategies could be employed: a simple partition of the data range into a large number of evenly sized hypercubes, for example, could also be used to discretize the data, although it runs the risk of having most or all of the data fall into a single hypercube. In practice, we have found *k-*means clustering to produce a reliable discretization of the data sets we have used.

### 4.3. On the Meaning of Scores

Scores are best thought of as a quantitative measure of how typical each segment of the sample is and how typical its surrounding segments are as compared to the training data. Raw scores exist on a scale that is unique to the model that produces them, and so raw scores cannot be compared across models. As a result, it will generally be preferable to use standardized *z*-scores. These are standardized against the distribution of scores from the training data: a *z*-score of 0.0 means that a sample scored as highly as the average sample from the training data; a *z*-score of 1.0 means that a sample scored one standard deviation higher than the average sample from the training data; a *z*-score of −1.0 means that a sample scored one standard deviation lower than the average sample from the training data. It is important to note that, although scores are related to probabilities, they are not and cannot be used as expressions of probability. Estimated *P* values, however, can also be calculated. These *P* values estimates are for the two-sided hypothesis that a random score for a sample from a pool like the training data would be more extreme than the current score, assuming a normal distribution of scores: users should verify that the scores produced by a model are approximately normally distributed before accepting the *P* values estimates.

### 4.4. Performance of WSPR Compared to SA+ as a Classifier

On the data sets used in this paper, the algorithm categorises samples as song or call correctly about 92% of the time. Compared to SA+, the algorithm makes about 1/3 as many assignment errors, a substantial improvement.

There are some caveats in the use of SA+ as a classifier, and some details that must be discussed regarding how it was used in this paper. SA+ was used to compare one single test sample against two single training samples, one from each category. In contrast, the algorithm described here compares a test sample against a digest of dozens or hundreds of training samples. A fairer comparison would involve using SA+ to compare a test sample against a large set of training samples and then using some averaging function to generate a score against each category. This is not typically how SA+ is used however, and SA+'s computationally intense method makes this infeasible: on a fast computer (2.6 GHz), comparing a one-second sample against 100 would take about 40 minutes. To classify a large group of samples in this manner, say 10 000, would take an unreasonable amount of time; hence we consider that the way in which SA+ was used here as a classifier is a fair reflection of how it would be used in practice.

### 4.5. Using Models to Estimate Stereotypy and Complexity

Aside from classification and general scoring tasks, the models produced by the WSPR algorithm can also be used to provide two measurements about the training data that may be of interest: stereotypy and complexity.

We propose that stereotypy can be thought of as a low degree of variance between samples. A low standard deviation of the scores of a sample set against a model provides the most direct measure based on this idea.

We can also define a measure of song repertoire complexity, by looking to the field of statistical complexity for inspiration. Measures of statistical complexity attempt to quantify the “structuredness” of a system or process. There is no consensus as to what exactly this means or how it could be best measured, but most proposals generally consider the number of parts in a system as well as the relationships between those parts.

**Figure 10:**
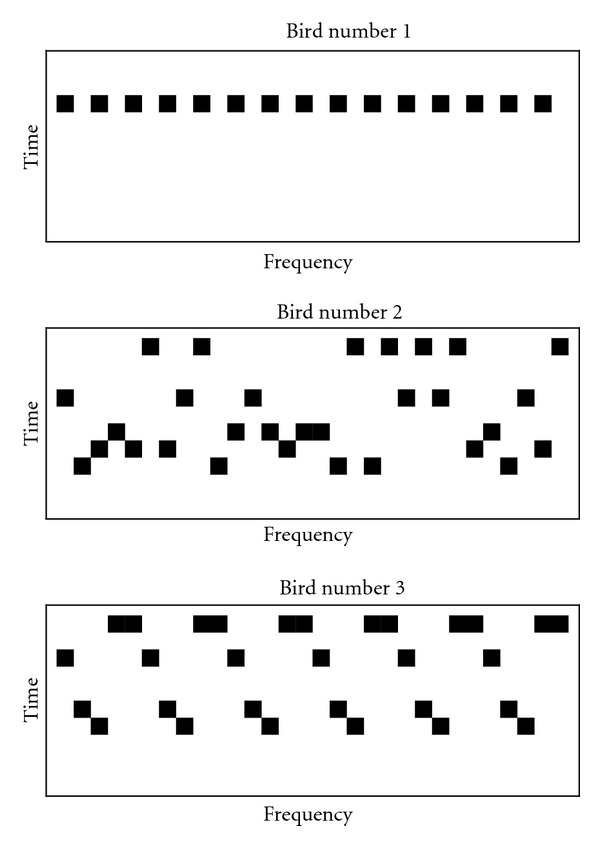
Hypothetical spectrograms for three birds. Bird number 1 has a song with a single note, bird number 2 has five notes but no pattern, and bird number 3 has five notes and a clear recurrent pattern

Let us consider this idea of complexity using several examples involving birdsong, illustrated with artificial examples in Figure 10. Bird number 1 has a one-note repertoire, and his song consists of repetitions of this note. He has a highly regular song, but it is very simple. His “system” has only a single “part” and we would suggest that his song is not complex.

Bird number 2 has a five-note repertoire; he sings randomly and each note is sung about 20% of the time. Although the song of bird number 2 has many parts, there are no relationships between these parts—each part is independent of the others, and no patterns emerge from his song beyond the individual notes. We would argue that, although the five notes make this bird's song more complex than bird number 1, he also has a fundamentally simple song structure.

Bird number 3 also has a five-note repertoire, but he sings with sequences of notes that appear regularly. Here, there are meaningful relationships between parts: some notes follow others at rates much higher than random chance. We would argue that this bird has what most observers would agree to be a more complex song structure.

Is there an existing measure that could be used for the purpose of measuring song complexity? Several measures of statistical complexity exist, for various problem domains [22–24], but the one that seems most relevant to birdsong is the measure of predictive information described by Bialek et al. [25]. To paraphrase, predictive information is how much more you know about the future states of a system upon learning about its past states. If combined with a measure of the number of different states (to prevent bird number 1 from receiving a high complexity score due to the high predictability of his song), predictive information is in accord with our intuitive ideas about birdsong complexity, wherein birds with regular patterns of notes make it possible to predict the note sequence, and the more extensive the patterns are, the more that can be predicted. This measure would also be in accordance with our intuitive notions about the complexity of the songs of the three birds discussed above.

**Figure 11:**
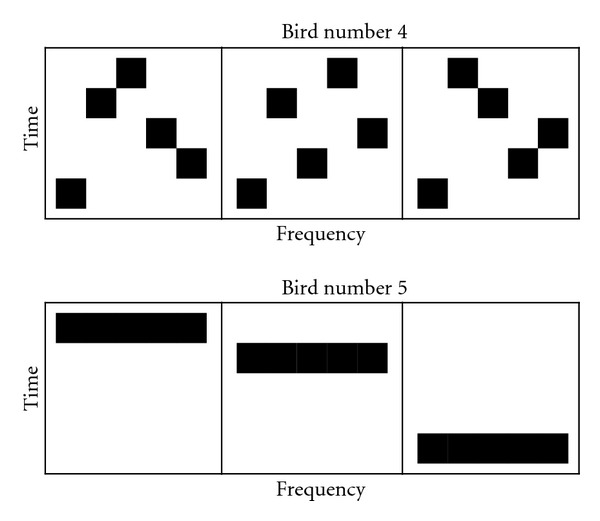
An illustration of why mutual information may not adequately capture intuitive notions of song complexity. Although the authors believe most people would agree that bird number 4 has a more complex song than bird number 5, the songs of both birds can have equal mutual information.

While predictive information seems like a good fit, Bialek et al. [25] use mutual information [26] as their underlying measure, and, under some circumstances, this may lead to counterintuitive results. For example, mutual information would consider a bird with five songs, each containing a different order of five different notes (bird number 4 in Figure 11), just as simple as a bird with five songs of one different note each (bird number 5 in Figure 11): both are equally predictable. For birdsong, a more appropriate underlying measure might be the Kullback-Leibler divergence [27], a measure of the difference between two probability distributions. The Kullback-Leibler divergence (KL divergence) would identify the former bird's repertoire as being more complex than the latter's. By using the KL divergence, we are subtly exchanging the idea of predictive information for a related but different one: the extent to which the past predicts changes in the future. It is our opinion that, of all the measures considered, the KL divergence most closely reflects intuitive ideas about song complexity. The measure we propose uses the data about sound distributions contained in the WSPR models to estimate the structural complexity of a sample set. It is essentially a mean of multiple KL divergences of distributions at different time intervals in the model. The exact formula can be found in the appendix.

An important consideration is that, for any measure, timing and the rate at which song features change are crucially important. On very short timescales, such as microseconds, a bird's song does not change much at all, and the correlation between past and present is total. On very long timescales, such as hours, the correlation between past and present singing behaviour is essentially zero. In between these extremes is a narrow range of timescales that optimally reveal the structure of the bird's song. We have not devised a satisfactory method for automatically identifying these optimal timescales and so can only recommend that care be taken in choosing timing parameters when attempting to measure song complexity using the method we propose.

### 4.6. Known Issues and Future Directions

One consideration when using WSPR is that background noise in recordings can be problematic: the algorithm does not distinguish background noise from vocalizations or any other noise of interest, and the background noise profile becomes built into the model. In the worst case, models built from samples with significant background noise may assign low scores to test samples simply because the background noise is different. To avoid this problem, noisy recordings should be denoised before either building a model or scoring against an existing model, or recording conditions should be managed to ensure a consistent level of background noise between training samples and test samples. With a sufficiently low level of background noise, WSPR models built from one dataset should be useable with datasets made in different recording environments, enabling the sharing of WSPR models between laboratories.

There are several important points to consider when using the measure of complexity we have provided. The measure is highly sensitive to the size of the sample set, so to make scores comparable across models, we recommend using exactly the same total length of sound to build each model. The measure can also be misled by extended periods of silence, especially if these frequently appear at the beginning or end of samples. To avoid this, we recommend trimming silent intervals from the ends of all samples. The model will also add implicit silence to the beginnings and endings of samples as necessary to make them at least as long as the window size of the model, so we recommend concatenating all samples that are less than twice as long as the window size before building a model.

WSPR bases its analysis on sound spectrograms, in part because this seems the most “natural” interpretation of the data, although many other song analysis tools have found success using higher-order measurements derived from the spectrograms [9–11]. One interesting extension to the work presented here would be to incorporate these higher-order measurements into the WSPR algorithm instead of or in addition to the spectrograms to see if scoring and classification accuracy could be improved further still.

## 5. Conclusions

In this paper, we have presented a novel method for comparing samples of birdsong against a larger set of samples, WSPR. WSPR is designed to cope with sample sets with low levels of stereotypy, an application that we feel no existing tool adequately addresses. We then extended this method to demonstrate a number of applications: classification problems, the original motivation behind the method's development; tracking song ontogeny; measuring song variability and complexity. We believe that the measure of birdsong complexity presented here represents the first effort of its kind.

Although the methods described in this paper are useable as they are, it is our hope that they may also serve as starting points for further discussion: in general, discussions about analyzing animal vocalizations and algorithms for doing so; in particular, discussions about what complexity means in the context of animal vocalization and how best to measure it.

## Figures and Tables

**Table 1:** Parameters used in all examples, unless specified otherwise.

Parameter	Value
STFT window width	500 samples (11.6 msec)
STFT step size	100 samples (2.9 msec)
STFT bandpass cutoffs	500 Hz–7500 Hz
Model window width	11 symbols (34.0 msec)
Number of power spectra clustered	7 500
Number of prototypes generated	120
Silence cutoff level	0.01 (arbitrary units)

**Table 2:** Summary of sample set sizes used to build and test models.

	Bird 1	Bird 2	Bird 3
Total samples	466	569	991
Manually classified as song	166	150	500
Manually classified as calls	300	419	491
Used as song training data	50	100	150
Used as call training data	100	150	150
Used as song testing data	116	50	350
Used as call testing data	200	269	341
Average sample length, song	4.5 seconds	1.1 seconds	0.4 seconds
Average sample length, call	1.8 seconds	1.1 seconds	0.8 seconds

## Appendix

### Details of the WSPR Algorithm

The preliminary step in building a model is to produce an encoding scheme for the sounds to be considered by the model. This is a form of vector quantization [28], in which the infinite variety of sounds is reduced to a finite set, or codebook, of representative sounds.

To generate a codebook of size *n* from a sample set of sounds:

- Convert each sample into power spectra via short-time Fourier transform.
- Take 100*n* random samples of power spectrum from all spectrograms.
- Cluster into *n* groups using *k*-means clustering.
- For each group:
  - Find the geometric mean of each frequency band across all samples in the cluster.
  - Normalise so that the mean spectra has a total power of 1.
  - The result is the prototypical spectral profile for the group.
  - Prepend a “null” spectrum of zeros to the beginning of the prototypes.

All samples must be transformed into a frequency-versus-time representation (a spectrogram) using a discrete-time short-time Fourier transform (STFT) [29]. Variations on the classical STFT are also acceptable; a STFT using a Gaussian window is used in our implementation of the algorithm. Phase information is discarded, as well as parts of the spectra outside a specific band of interest, that is, below 500 Hz or above 7500 Hz.

In cases where one wants to build two or more models from the same underlying encoding, the samples used to generate the codebook should be taken from the joint sample set of the two models.

Encoding Samples Having constructed a codebook, all samples must now be encoded. To encode a sample:

- Convert via STFT to power spectra.
- For each power spectrum *s* in the spectrogram:
  - If the total power in *s* is below the cutoff threshold, emit a zero (the null spectrum).
  - Otherwise, examine the codebook, *P*, to find the spectrum, *p*, where the root mean square deviation between *s* and *p* is minimized.
  - Emit the index number of *p*, that is, if *p* is the 3rd spectrum in the codebook, then emit a 3.
  - The sequence of emitted numbers is the encoding of the sound.

Constructing the Model Perform the following steps to construct a model using the encoded samples:

- Choose a sliding window length, *w*. Define *a* as the middle (anchor) position of the window.
  (A.1) $$a=\left\lfloor \frac{w+1}{2}\right\rfloor .$$
- Create an array *M* of dimension *n* × *w* × *n* and a vector *T* of length *n*. Initialise all values in *M* and *T* to 0. For every encoded sample *e*, create a set *R* of all possible subsequences of *e* of length *w*.
- For each *r* in *R*, perform the following:
  (A.2) $$\begin{array}{c} T_{r_{a}}⟵T_{r_{a}}+1,\\ M_{r_{a},j,r_{j}}⟵M_{r_{a},j,r_{j}}+1\;\forall j\in \left\{1,\ldots ,w\right\}, \end{array}$$
  where *r*_*a*_ is the symbol at the anchor position in *r*.
- Create an array *M**, same size as *M*; vector *T**, same size as *T*.
- For *i* in 1,…, *n*, perform the following computation:
  (A.3) $$T_{i}^{*}=\frac{T_{i}}{\sum_{j=1}^{n}T_{j}}.$$
- For *j* in 1,…, *w*, *k* in 1,…, *n*, perform the following computation:
  (A.4) $$M_{i,j,k}^{*}=\frac{M_{i,j,k}}{\sum_{l=1}^{n}M_{i,j,l}}.$$

The tuple of *W* = (*P*, *T**, *M**) constitutes the constructed model, where *r*_*a*_ is the symbol at the anchor position in *r*.

Scoring a Sample against the Model Finally, we must be able to compute a score of a test sample against a model. Perform the following steps to score a test sample against a model *W*:

- Convert the sample to a spectrogram via STFT.
- Encode the sample as described previously, creating encoding *e*.
- Create a set *Q*, containing every subsequence of *e* of length *w*.
- For each *q* in *Q*, calculate the following:

(A.5) $$\zeta \left(q,M^{*}\right)=T_{q_{a}}\left({\left(\overset{w}{\underset{j=1}{\prod }}M_{q_{a},j,q_{j}}^{*}\right)}^{1/w}\right).$$

- The score of the sample is

(A.6) $$Z\left(Q;W\right)=\ln\left({\left(\overset{\text{length}(Q)}{\underset{i=1}{\prod }}\zeta \left(q_{i},M^{*}\right)\right)}^{1/\text{length}(Q)}\right).$$

*Z*(*Q*; *W*) is the non-standardized (“raw”) score of *Q* against the model *W*.

Standardization of Scores and Estimation of *P-*Values After the model is built, every sample in the training set is scored against the model. The mean (*μ*) and standard deviation (*σ*) of these raw scores are calculated and stored along with the model. When a test sample is scored against the model, its normalised *z*-score can be computed as

(A.7) $$Z^{*}\left(Q;W\right)=\frac{\mu -Z\left(Q;W\right)}{\sigma }.$$

To calculate an estimated *P* value, the CDF of a normal distribution with mean *μ* and standard deviation *σ* is used to determine the proportion of the distribution that is more extreme than the test score.

Classification Using Multiple Models Suppose we have *x* known classes (1,2, 3,…) to which we wish to assign samples and training data sets {*S*_1_, *S*_2_,…, *S*_*x*_}. We begin by building a joint set of prototypes for all samples from {*S*_1_, *S*_2_,…, *S*_*x*_} as described previously. Then, for each set of samples *S*_*i*_, we build a model *W*_*i*_ using the algorithm described previously. We calculate the means and standard deviations for each sample set against each model, producing a *x* × *x* table for each statistic:

(A.8) $$\begin{array}{cc} & \mu_{i,j}=\text{mean}\left(Z\left(s;W_{j}\right)\right),\\ & \sigma_{i,j}=\mathrm{sd}\left(Z\left(s;W_{j}\right)\right), \end{array}\;\forall s\in S_{i},\;j\in \left\{1,\ldots ,x\right\}.$$

The tuple (*P*, {*W*_1_, *W*_2_,…}, *μ*, *σ*) constitutes the classifier. When a sample *Q* is submitted for classification, we calculate the raw score *Z*(*Q*; *W*_*j*_) for all *W*_*j*_. Then, we calculate the typicality of *Z*(*Q*; *W*_*j*_) relative to each training set *S*_*i*_:

(A.9) $$Y\left(Q,i,j\right)=\frac{Z\left(Q;W_{j}\right)-\mu_{i,j}}{\sigma_{i,j}}\;\forall j\in \left\{1,\ldots ,x\right\}.$$

Finally, we calculate the “atypicality” (or deviation of typicality) for each class as

(A.10) $$A\left(Q,j\right)=\sqrt{\overset{x}{\underset{j=1}{\sum }}Y{\left(Q,i,j\right)}^{2}.}$$

The *i* for which *A*(*Q*, *i*) is lowest is the class to which *Q* is assigned. This method works not by assigning a sample to the model for which it is most typical but by assigning a sample to the class of samples whose scores are most similar across all the models; in practice this seems to provide an improvement in accuracy.

Calculating the Complexity of a Model Take *T** and *M** from a model *W*. Recall that *T** is of length *n* and *M** is of dimension *n* × *w* × *n*. Then,

(A.11) $$\begin{array}{cc} & \Gamma \left(T^{*},M^{*},k\right)\\ & \;=\left\{T_{i}^{*}M_{i,j,k}^{*}\right\}\\ & \;=\left\{\gamma_{1},\gamma_{2},\ldots ,\gamma_{n\times n}\right\}\;\forall i\in \left\{1,\ldots ,n\right\},\;j\in \left\{1,\ldots ,n\right\}. \end{array}\;$$

That is, if *M*_*i*,*j*,*k*_* is the probability of seeing symbol *j* at position *k* given that symbol *i* is at the anchor position, then Γ(*T**, *M**, *k*) for each *k* is the set of all elements *M*_*i*,*j*,*k*_* for that value of *k*, each multiplied by the probability of seeing symbol *i*. Given the definition of the Kullback-Leibler divergence as

(A.12) $$D_{\text{KL}}\left(P,Q\right)=\sum_{i}P\left(i\right)\text{lo}{\text{g}}_{2}\frac{P\left(i\right)}{Q\left(i\right)},$$

then our measure of song complexity is calculated as follows:

(A.13) $$\begin{array}{cc} & C_{\text{song}}\left(T^{*},M^{*},x,y\right)\\ & \;=\frac{1}{y-x+1}\overset{y}{\underset{i=x}{\sum }}D_{\text{KL}}\left(\Gamma \left(T^{*},M^{*},a+1\right),\Gamma \left(T^{*},M^{*},a+i+1\right)\right), \end{array}$$

where *M** and *T** are the components of a model *W*, *a* is the anchor position of that model, and *x* and *y* are the start and end of a range of positions in the model forward of the anchor position for which the complexity is to be calculated.
